# Microbial detection in seroma fluid preceding the diagnosis of breast implant-associated anaplastic large cell lymphoma: a case report and review of the literature

**DOI:** 10.1080/23320885.2019.1593846

**Published:** 2019-04-02

**Authors:** A. Fricke, J. A. Wagner, J. Kricheldorff, C. Rancsó, U. Von Fritschen

**Affiliations:** a Department of Plastic and Aesthetic Surgery, Hand Surgery, HELIOS Hospital Emil von Behring, Berlin, Germany;; b Department of Plastic and Aesthetic Surgery, HELIOS Hospital Berlin-Buch, Germany;; c Department of Pathology, HELIOS Hospital Emil von Behring, Berlin, Germany

**Keywords:** Breast implant-associated anaplastic large cell lymphoma, late-onset seroma, grouping, breast implant

## Abstract

The Breast Implant-Associated Anaplastic Large Cell Lymphoma (BIA-ALCL) represents a topic of great concern. We report the case of a patient with late-onset seroma, who was initially diagnosed with an implant-related infection of the breast due to microbial detection in the seroma fluid, thus delaying the diagnosis of BIA-ALCL.

## Introduction

Breast Implant-Associated Anaplastic Large Cell Lymphoma (BIA-ALCL), which has been declared a provisional new disease entity, was estimated to occur with a relative risk of 421.8 in women with breast implants; while the number of women with breast implants needed to cause one BIA-ALCL before the age of 75 years was 6920 [1]. Although the MBN 2016 Aesthetic Breast Meeting BIA-ALCL Consensus Conference Report states that a statistically significant association between BIA-ALCL and any implant, patient or surgery characteristic has not been found when analysing 126 peer-reviewed publications on BIA-ALCL [2], BIA-ALCL has been described to be more common in association with textured implants [3].

In spite of the fact that periprosthetic minor fluid accumulation is not unusual, larger late-onset seromas occur only in 0.1–0.2% of patients following implantation of textured implants [4], approximately 10% of which Di Napoli et al. report to be associated with BIA-ALCL [5,6]. Furthermore, late-onset, rapid progressive peri-prosthetic seroma is present in 70-90% of BIA-ALCL cases [7–9]. Thus, any delayed seroma occuring more than one year after implantation not explainable by infection or trauma [2] might potentially be associated with BIA-ALCL and should therefore be investigated through fine-needle aspiration, flow cytometry, and CD30/ALK immunohistochemistry [2,6]. Of note, the pathologist should ideally be supplied with >100 mL of seroma fluid [6]. Repeated aspirations acquiring small volumes should be avoided since this can potentially result in “tumor dilution” [10] and thus in insufficient cellular material for immunohistochemical analysis or flow cytometry [10,11], possibly leading to false-negative diagnosis of BIA-ALCL. Ultrasound and magnetic resonance imaging are used for diagnosis of BIA-ALCL, further assessing the presence of a tumour mass or lymph node enlargement. For staging, the suggested imaging method is positron emission tomography and computed tomography (PET/CT) [6,12].

## Case report

A 56 year-old woman presented with swelling of the left breast. Seven years before, bilateral subcutaneous mastectomy with expander reconstruction was performed due to bilateral breast cancer followed by radiotherapy and chemotherapy. Although recommended, the patient decided against bilateral autologous tissue reconstruction. Thus, one year later, implant reconstruction was carried out, using textured silicone implants (Inspira N-TRM 600, Allergan®). Due to threatening skin perforation, revision surgery was performed 3 months later, replacing the left implant (Allergan® Inspira N-TSM 520) and stabilising the skin with porcine acellular dermal matrix (Strattice^TM^, Allergan®). Again, autologous tissue reconstruction was refused by the patient.

Five and a half years later, in 2016, the patient presented at her gynecologist’s, reporting swelling of the left breast without any further signs of infection. Sonographically-guided sterile puncture of the late-onset seroma of the left breast was carried out several times. Since microbiological analysis of the seroma fluid detected the growth of Staphylococcus epidermidis, implant-related infection of the breast was suspected and oral antibiotic treatment was initiated. The swelling initially subsided; however, it recurred several months later. Only then, immunohistochemical analysis of the seroma fluid was performed, revealing CD30 – positivity. The case was then discussed in an interdisciplinary tumour conference, recommending an oncohematological consultation including staging. Although PET/CT has been suggested for staging of BIA-ALCL [6,12], PET/CT was not performed since it is not covered by the German health insurance. Magnetic resonance imaging and computed tomography of the abdomen and chest did not show a suspicious tumour mass, enlarged regional lymph nodes or signs of a systemic disease.

**Figure 1.**
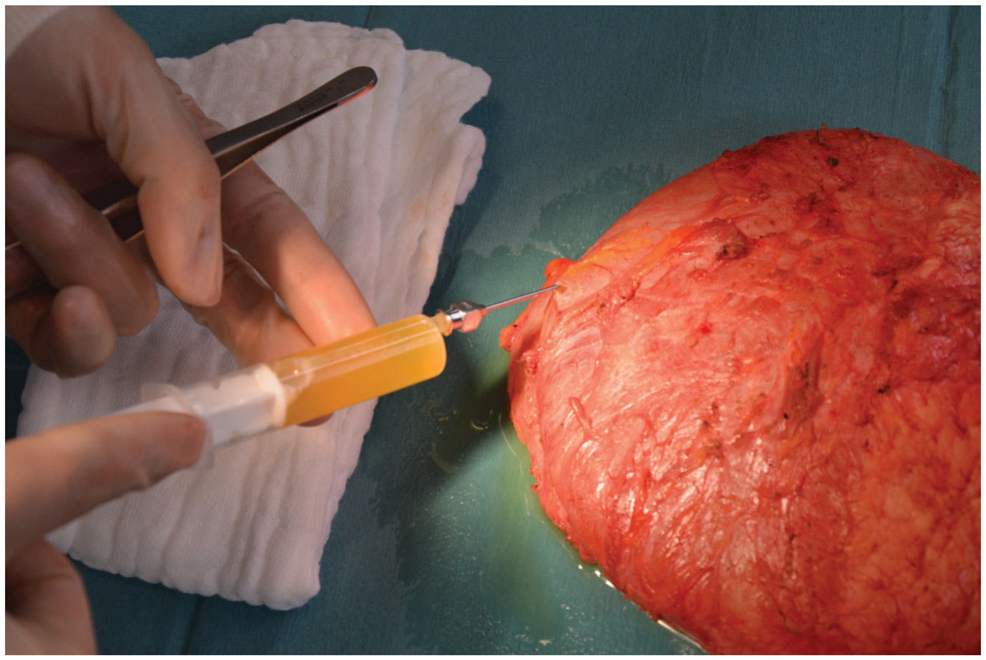
Aspiration of intracapsular seroma fluid after explantation of the intact capsule containing the implant.

**Figure 2.**
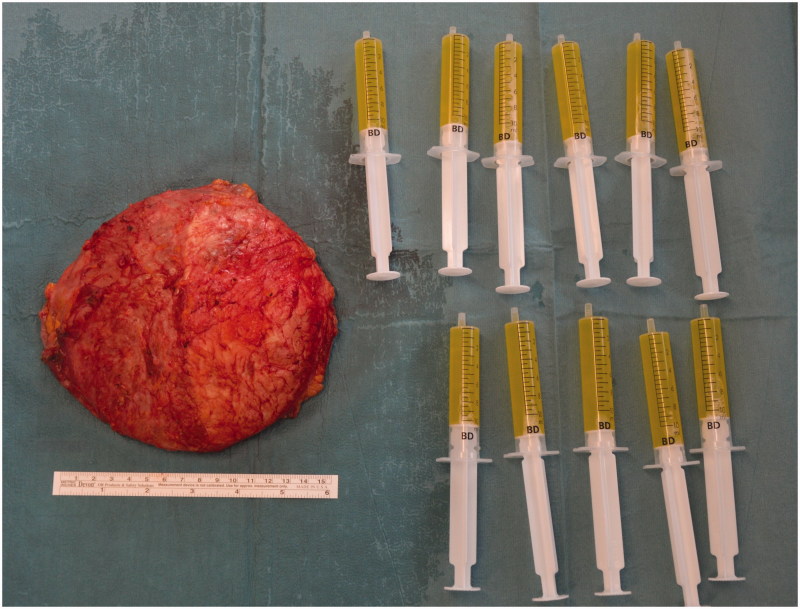
Intracapsular seroma fluid.

**Figure 3.**
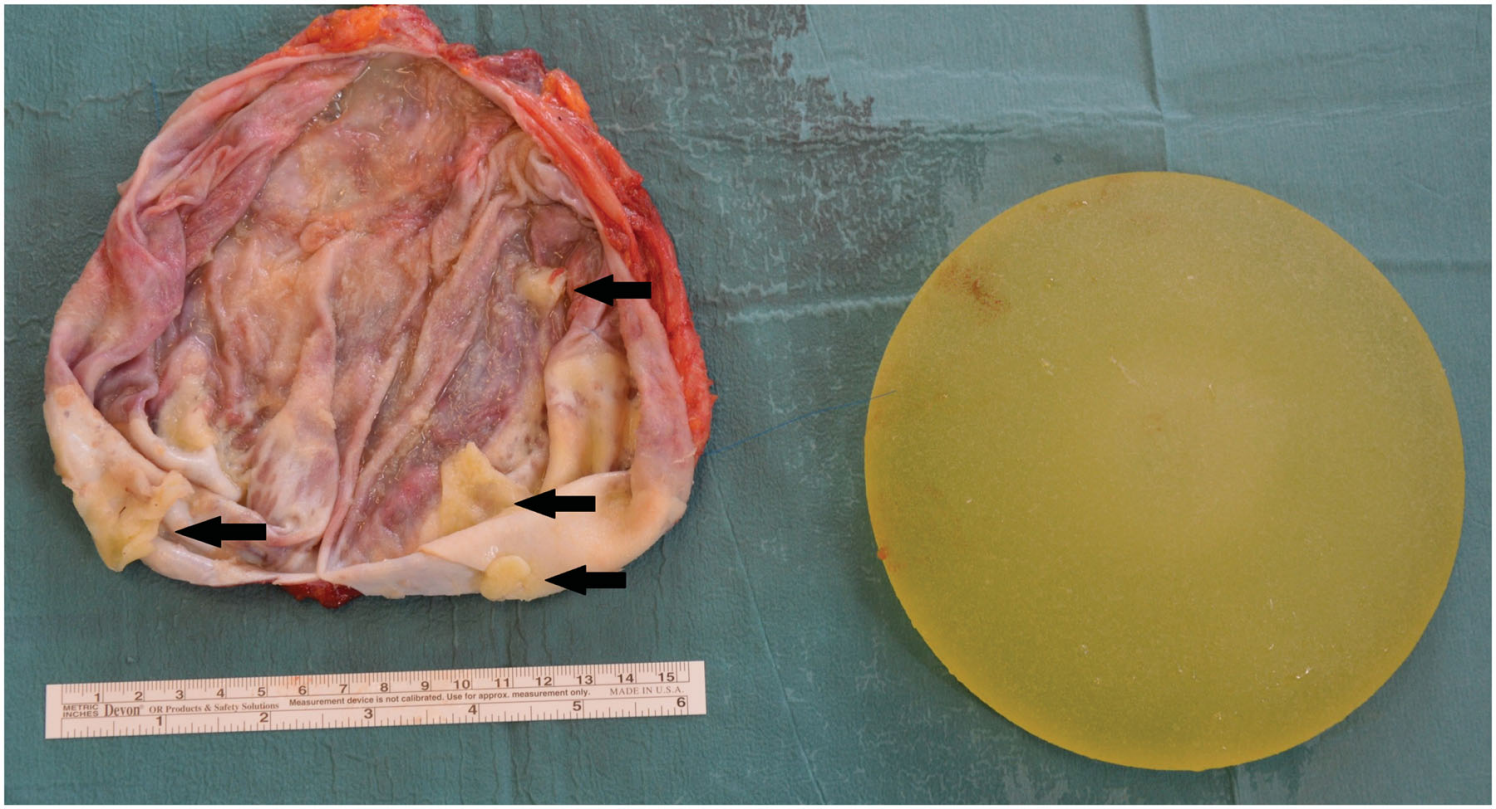
Inner surface of the capsule (left) and intact mammary implant (right). The histopathological analysis including immunohistochemistry showed conglomerates of fibrin deposits and CD30 positive and ALK negative tumour cells on the inner surface of the capsule (black arrows).

**Figure 4.**
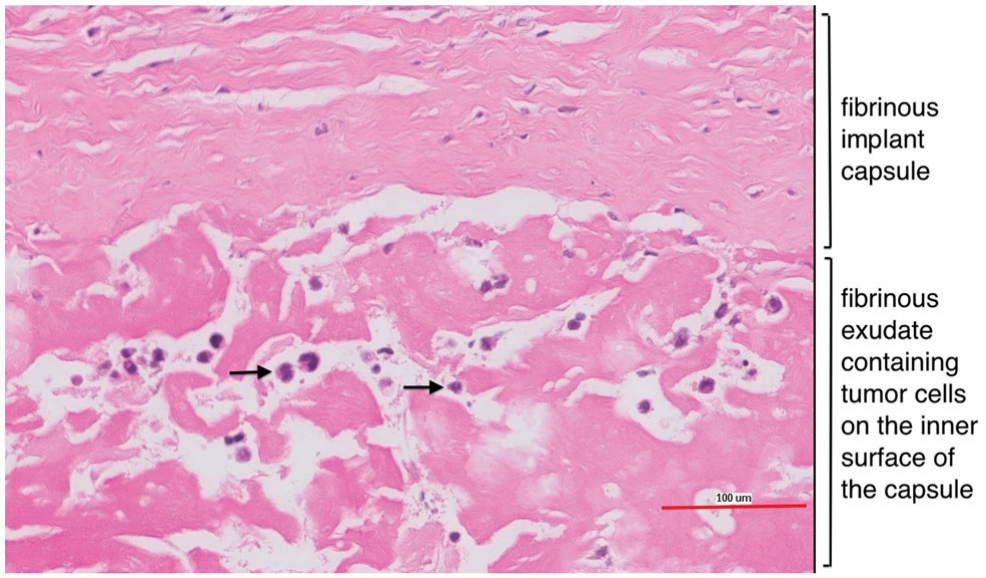
Histological image of the fibrinous implant capsule and the fibrinous exudate containing anaplastic tumour cells on the inner surface of the capsule. Black arrows = anaplastic tumour cells.

**Figure 5.**
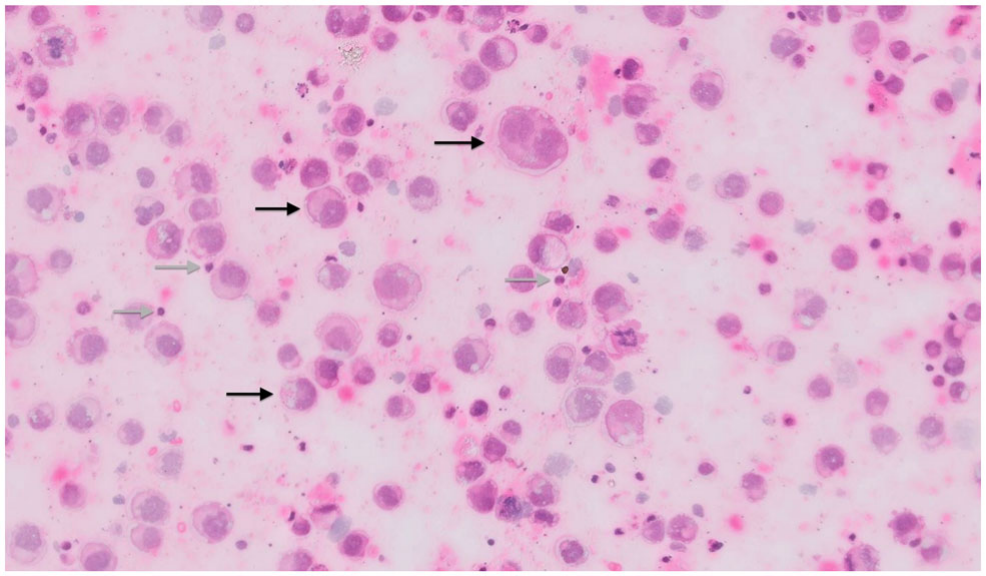
Cytological image of the neoplastic seroma fluid containing numerous highly atypical large anaplastic tumour cells characteristic for anaplastic large-cell lymphoma (ALCL) as well as scant inflammatory cells, predominantly lymphocytes (Hematoxylin and eosin stain; black arrows = anaplastic tumour cells; grey arrows = lymphocytes).

**Figure 6.**
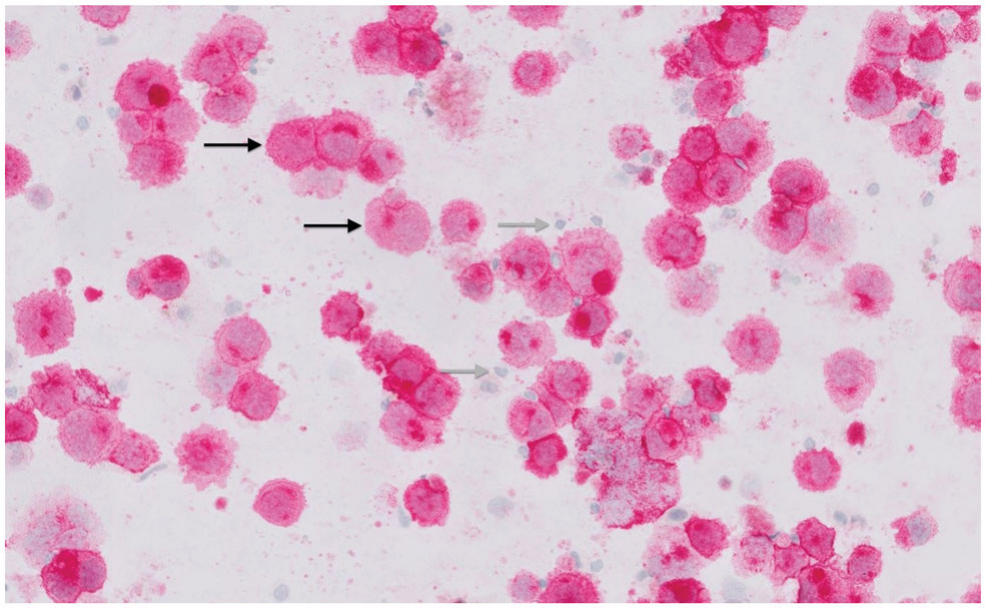
Cytological image of the neoplastic seroma fluid containing numerous highly atypical large anaplastic tumour cells characteristic for anaplastic large-cell lymphoma (ALCL) as well as scant inflammatory cells, predominantly lymphocytes (CD30 stain; black arrows = anaplastic tumour cells; grey arrows = lymphocytes).

Thereafter, the patient presented at our department. We performed a removal of the complete capsule containing the implant, acquiring the intracapsular seroma fluid by aspiration after explantation ( Figures 1–3). A smooth surface implant was used as a spacer until definite reconstruction by autologous tissue transfer. Due to the risk of an incidental contralateral BIA-ALCL [6,13], the removal of the contralateral implant was recommended; however, the patient preferred not to have her contralateral implant removed until definite reconstruction by autologous tissue transfer. The histopathological analysis including immunohistochemistry showed a fibrinous exudate containing CD30 positive and ALK negative tumour cells on the inner surface of the capsule (Figure 3, black arrows indicating fibrin deposits; and Figure 4; black arrows indicating anaplastic tumour cells), thus confirming the diagnosis of an *in situ* BIA-ALCL. Figure 5 (Hematoxylin and eosin stain) and Figure 6 (CD30 stain) show the neoplastic seroma fluid, which exhibits numerous highly atypical large anaplastic tumour cells characteristic for anaplastic large-cell lymphoma (ALCL) as well as scant inflammatory cells, predominantly lymphocytes (black arrows = anaplastic tumour cells; grey arrows = lymphocytes). Microbiological analysis of the neoplastic fluid was negative at the time of surgery; however, the patient had priorly been treated with oral antibiotics for several weeks.

## Discussion

We report a case of a patient with late-onset seroma of the breast after implant reconstruction, who was initially diagnosed with an implant-related infection of the breast due to the detection of Staphylococcus epidermidis in the seroma fluid. After diagnosis of BIA-ALCL, almost seven months later, the complete capsule containing the implant was removed [14].

In this context, Di Napoli et al. report microbial detection in 23,8% (5 of 21) of all reactive late seromas of the breast which were sent for culture; however, they do not specify whether pathogen growth was detected in the seroma fluid of BIA-ALCL patients [5]. Most studies do not mention if microbial analysis of the seroma fluid of BIA-ALCL patients was carried out [2,6–8,12]. However, Hu et al. reported the detection of bacterial biofilm in both BIA-ALCL samples as well as non-tumour capsule samples, showing a predominance of *Ralstonia spp.* in BIA-ALCL samples and contralateral capsule samples [15]. The authors argue that the presence of a biofilm might trigger the formation of BIA-ALCL due to chronic bacterial antigen stimulation [15]. Interestingly, however, they showed that in capsules from the contralateral breast of BIA-ALCL patients, a significantly lower number of bacteria was detected, suggesting that the activation of lymphocytes correlates with the bacteria load [15]. The fact that textured implants were found to present a higher bacteria load [16,17] might thus provide an explanation for the higher incidence of BIA-ALCL in patients with textured implants [3,15]. However, the ‘subclinical infection hypothesis’ described by Hu et al. [15] has been questioned by several authors, including Santanelli di Pompeo et al. [18]. The authors state that it remains to be elucidated why, amongst other things, *Ralstonia spp.* has also been detected in non-ALCL capsule samples [15] as well as why biofilm is present on all types of breast implants [16,17] although ALCL is associated predominantly with textured implants [3,18]. Nevertheless, one has to consider that thicker biofilms have been found on implants with rougher surface textures [17].

Swanson [19] underlines the fact that BIA-ALCL samples may be negative for bacteria and bacterial growth has also been found in control samples [15,19]. Thus, Swanson questions the ‘subclinical infection hypothesis’, stating that the effect of texturing is much more likely to trigger BIA-ALCL. The author therefore suggests to avoid textured implants rather than searching for microbial growth in BIA-ALCL samples [19].

Thus, one needs to consider that although bacterial biofilm is often present when examining the implant capsule of BIA-ALCL samples [15], one cannot be certain that biofilm formation causes BIA-ALCL. The aetiology of BIA-ALCL therefore seems more likely to be multifactorial.

Nevertheless, microbial detection in seroma fluid might detract the attending physician’s attention from the diagnosis of BIA-ALCL, thus delaying diagnosis and timely treatment.

In conclusion, relevant late effusions should always be sent for both microbial analysis and cytological examination. If the amount of fluid available is not sufficient for both microbial analysis and cytological examination, priority should be given to pathological examination in order to preclude malignancy, further providing an indication of the presence of bacterial infection or implant damage.
